# Comparative Analyses between Skeletal Muscle miRNAomes from Large White and Min Pigs Revealed MicroRNAs Associated with Postnatal Muscle Hypertrophy

**DOI:** 10.1371/journal.pone.0156780

**Published:** 2016-06-02

**Authors:** Xihui Sheng, Ligang Wang, Hemin Ni, Lixian Wang, Xiaolong Qi, Shuhan Xing, Yong Guo

**Affiliations:** 1 Animal Science and Technology College, Beijing University of Agriculture, Beijing, 102206, China; 2 Beijing Key Laboratory of Traditional Chinese Veterinary Medicine, Beijing University of Agriculture, Beijing, 102206, China; 3 Key Laboratory of Farm Animal Genetic Resources and Germplasm Innovation of Ministry of Agriculture of China, Institute of Animal Science, Chinese Academy of Agricultural Sciences, Beijing, 100193, China; Wageningen UR Livestock Research, NETHERLANDS

## Abstract

The molecular mechanism regulated by microRNAs (miRNAs) that underlies postnatal hypertrophy of skeletal muscle is complex and remains unclear. Here, the miRNAomes of *longissimus dorsi* muscle collected at five postnatal stages (60, 120, 150, 180, and 210 days after birth) from Large White (commercial breed) and Min pigs (indigenous breed of China) were analyzed by Illumina sequencing. We identified 734 miRNAs comprising 308 annotated miRNAs and 426 novel miRNAs, of which 307 could be considered pig-specific. Comparative analysis between two breeds suggested that 60 and 120 days after birth were important stages for skeletal muscle hypertrophy and intramuscular fat accumulation. A total of 263 miRNAs were significantly differentially expressed between two breeds at one or more developmental stages. In addition, the differentially expressed miRNAs between every two adjacent developmental stages in each breed were determined. Notably, ssc-miR-204 was significantly more highly expressed in Min pig skeletal muscle at all postnatal stages compared with its expression in Large White pig skeletal muscle. Based on gene ontology and KEGG pathway analyses of its predicted target genes, we concluded that ssc-miR-204 may exert an impact on postnatal hypertrophy of skeletal muscle by regulating myoblast proliferation. The results of this study will help in elucidating the mechanism underlying postnatal hypertrophy of skeletal muscle modulated by miRNAs, which could provide valuable information for improvement of pork quality and human myopathy.

## Introduction

Skeletal muscle development is critical for growth rate and pork production in pig (*Sus scrofa*). Understanding the complex mechanism underlying skeletal muscle development will be beneficial for genetic improvement of lean meat percentage and pork quality. Skeletal muscle growth is also essential for animal and human health because it comprises 40–60% of body weight and is important for locomotion and metabolic homeostasis. Pig has been recognized as a suitable model for research into various aspects of human diseases, such as muscular atrophy, obesity, and diabetes, because it is closer evolutionarily to humans than mice [1].

Skeletal muscle development is a complex process, encompassing embryonic myogenesis and postnatal muscle growth. Embryonic myogenesis in pig has three successive waves [2,3] which constitute the origin of the different types of muscle fibers. Following these three waves of myogenesis, the total number of fibers is fixed, which is a key determinant of postnatal muscle growth. Consequently, postnatal muscle growth relies largely on satellite cell proliferation and protein turnover which leading to muscle hypertrophy [4]. Protein turnover occurs when the overall rates of protein synthesis exceed the rates of protein degradation. Two major signaling pathways control protein synthesis: the insulin-like growth factor 1–phosphoinositide-3-kinase/Akt (protein kinase B)–mammalian target of rapamycin (IGF1—Akt—mTOR) pathway, which acts as a positive regulator [5]; and the myostatin—Smad2/3 pathway, which acts as a negative regulator [6, 7]. Satellite cell proliferation is also important for postnatal muscle growth. It was reported previously that destroying the cell proliferative capacity of muscles by gamma irradiation prevented compensatory hypertrophy of the overloaded *extensor digitorum longus* muscle in rats [8]. Moreover, satellite cell differentiation was found to modulate myofiber growth by regulating the number of satellite cells capable of proliferating [9].

MicroRNAs (miRNAs) are small non-coding RNAs that negatively modulate gene expression through translational repression or by causing degradation and deadenylation of target mRNAs [10, 11]. Previous studies have identified many miRNAs implicated in skeletal muscle hypertrophy [12]. For example, muscle-specific miRNAs (myomiRs), such as miR-1, miR-133a/b, miR-206, and miR-486, were shown to be involved in the regulation of skeletal muscle hypertrophy by modulating the IGF-1–Akt pathway and myostatin signaling pathway [13–17]. However, information about the molecular mechanisms underlying the regulation of skeletal muscle hypertrophy by miRNAs is still limited.

Large White (LW; commercial breed) pig is characterized by high lean meat percentage, fast-growing muscle, and high body weight. In contrast, Min pig (M, indigenous breed of China) is characterized by high intramuscular fat content, slow-growing muscle, and low body weight. The different characteristics of muscle between the two breeds make them potentially good models for studying the mechanism underlying skeletal muscle development. Previous studies have focused mainly on myogenesis at different stages of fetal development [18–20]. In this study, we collected the *longissimus dorsi* muscle from LW and M pigs at five postnatal stages, namely 60, 120, 150, 180, and 210 days postnatal (dpn), and analyzed the miRNAomes by Illumina sequencing. The results will provide comprehensive information about the miRNAs associated with postnatal hypertrophy of skeletal muscle in pig.

## Materials and Methods

### Ethics statement

Animal welfare and experimental procedures were carried out in accordance with the Guide for the Care and Use of Laboratory Animals (Ministry of Science and Technology of China, 2006), and were approved by the animal ethics committee of Beijing University of Agriculture.

### Animals and tissue collection

The 15 male LW pigs and 15 male M pigs that were used in this study were housed at the Animal Science Academy, Jilin Academy of Agricultural Sciences, China, under similar environmental and nutritional conditions. The temperature and relative humidity of the pig house were 15–22°C and 65 ± 10%, respectively. Pigs were allowed free choice access to food and tap water. The basic diet was formulated as recommended by the National Research Council (1998). At 60, 120, 150, 180, and 210 dpn, three pigs per breed were selected randomly and slaughtered in a local slaughter house. Briefly, the pigs to be slaughtered were relaxed first by a warm shower. Then the pigs were stunned by carbon dioxide (CO_2_), hanged and slaughtered. The *longissimus dorsi* muscles near the last third or fourth rib were dissected rapidly from the carcasses and submerged in RNAlater (Qiagen, Hilden, Germany), after which they were stored at −20°C.

### Small RNA library construction and Illumina sequencing

Total RNA from 30 pigs of two breeds was isolated using TRIzol reagent (Invitrogen, CA, USA) respectively and analyzed on a Bioanalyzer 2100 (Agilent, CA, USA) to ensure adequate quality and quantity of RNA. The total RNA samples from three pigs per breed at same postnatal stage were pooled and prepared for small RNA (sRNA) library construction. A total of ten sRNA libraries of two breeds at five postnatal stages were constructed respectively. In brief, 15% denaturing polyacrylamide gel electrophoresis (PAGE) was used to purify the sRNAs with lengths of 18–30 nucleotides (nt) from 10 μg of total RNA. The sRNAs were ligated with proprietary adapters and amplified by RT-PCR. The amplification products were then separated on a PAGE gel and used for sequencing on a HiSeq 2000 analyzer (Illumina, CA, USA).

### Analysis of sequencing data

First, low quality reads and adapter sequences were removed to obtain clean reads. Then the clean data were mapped to the pig reference genome (http://www.ensembl.org/Sus_scrofa/Info/Index, Sscrofa 10.2) using the Burrows-Wheeler alignment (BWA) mapping tool [21] and blasted against the Rfam database (http://rfam.sanger.ac.uk, Rfam 12.0) [22] to detect other non-coding RNAs. After discarding transfer RNAs (tRNAs), ribosomal RNAs (rRNAs), small nucleolar RNAs (snoRNAs) and small nuclear RNA (snRNAs), the reads that aligned perfectly (no mismatches) to the pig genome were used for further analysis.

### MiRNAs identification and prediction

All the reads that remained after the above analyses were searched against miRBase (http://www.mirbase.org/; Release 21: June 2014) [23] to identify known porcine miRNAs. Unannotated sequences that did not match any of the above databases were analyzed by Mireap (http://sourceforge.net/projects/mireap/) to predict candidate novel miRNAs. The ability of the predicted miRNAs to form hairpin structures was estimated using RNAfold [24]. Cluster 3.0 and TreeView were used to cluster and analyze the known and predicted miRNAs in ten sequence libraries of two breeds.

### Identification of differentially expressed miRNAs

Differentially expressed (DE) miRNAs were detected using the edgeR package [25]. Normalization factors were computed using the TMM technique, after which tag-wise dispersions were calculated and subjected to an exact test. The resulting *P* values were subjected to Benjamini—Hochberg multiple testing correction to derive false discovery rates (FDRs). Only miRNAs with a FDR < 0.01 and folder change > 2 were considered for further analysis.

### Targets prediction, and gene ontology and KEGG pathway analyses

To understand the potential functions of the DE miRNAs, we used TargetScan (http://www.targetscan.org/) [26] and miRDB (http://www.mirdb.org/miRDB/policy.html) [27] to predict target genes for the known and novel miRNAs. Because *S*. *scrofa* genes have not been included in the current version of these databases, our predictions based on the human miRNA: mRNA interactions. The predicted target genes were subsequently submitted to DAVID (https://david.ncifcrf.gov/home.jsp) [28] for gene ontology (GO) and Kyoto Encyclopedia of Genes and Genomes (KEGG) pathway analyses with the following parameters: Count = 2 and EASE = 0.1.

## Results

### Overview of sequencing data

**Fig 1:**
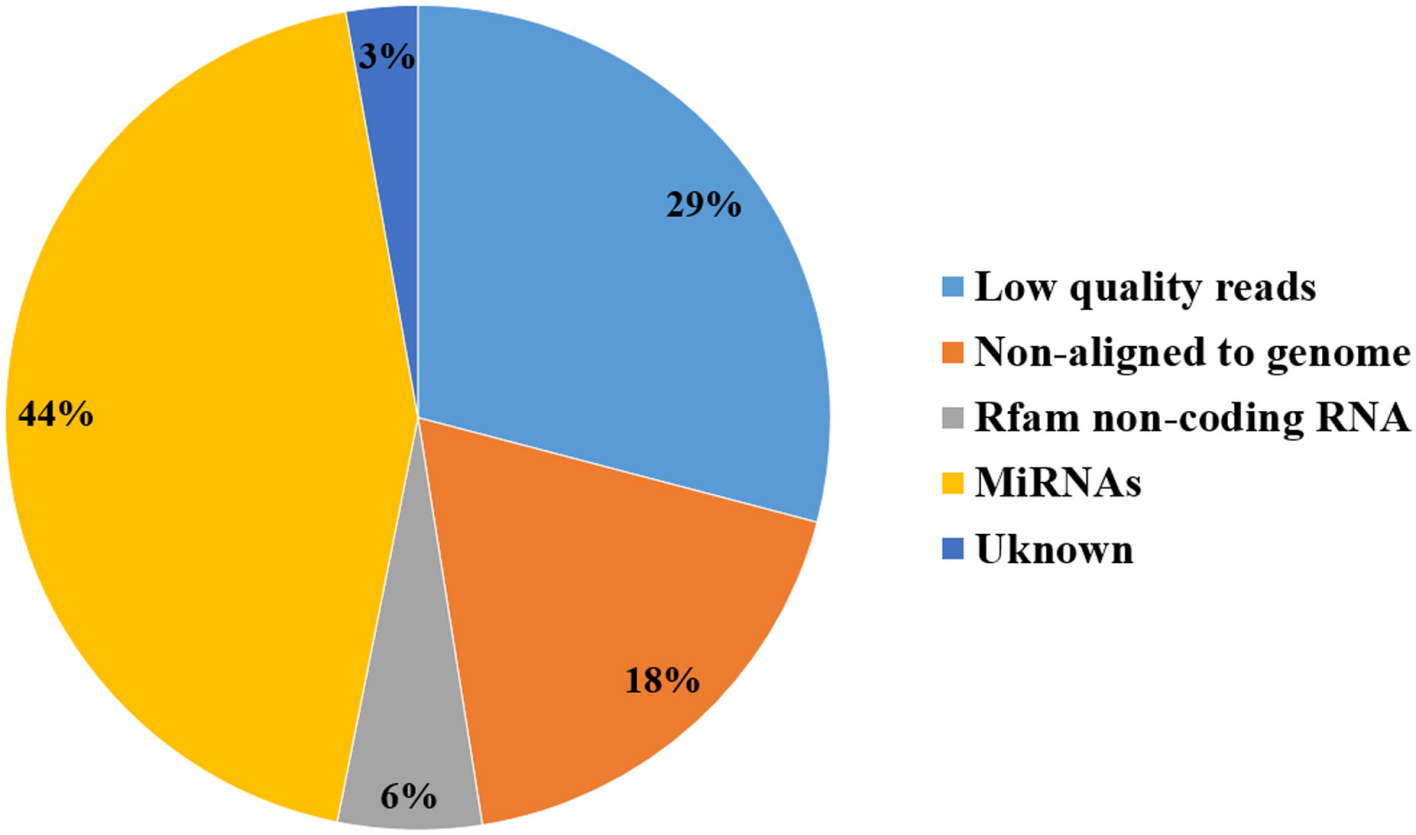
Overview of the small RNA sequences in the 10 libraries. Each number in the pie chart represents the percentage of reads of that category in the total small RNA sequences.

**Fig 2:**
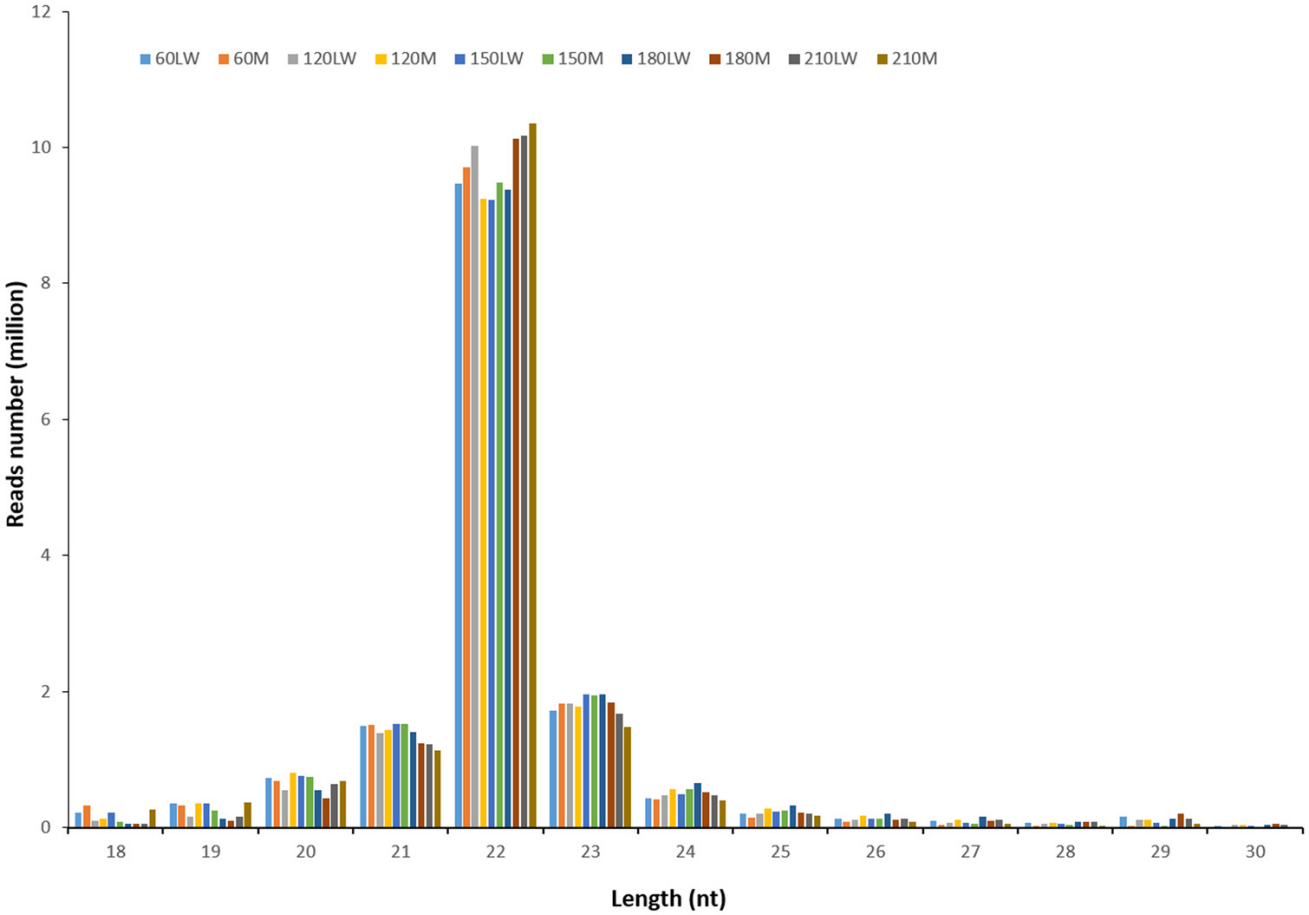
Length distribution of the small RNA sequences. LW, Large White pig; M, Min pig.

A total of about 212.69 million (M) raw reads (10.63 Gb) were obtained from the ten sRNA libraries, giving an average of 21.27 M reads (1.06 Gb) per library. After eliminating adaptor sequences and low quality reads, 150.83 M clean reads (with an average of 15.08 M reads per library) were retained, which corresponded to 70.9% of the total reads (Fig 1). The sequence length ranged from 18 to 30 nt, the 22 nt reads had the highest abundance, followed by 23 nt and 21 nt reads (Fig 2), which is in accordance with the known 21–23 nt range for miRNAs [10]. About 74.02% of the high quality sequences perfectly matched the pig reference genome, and 11.99 M reads (7.95% of the high quality reads) matched the non-coding RNAs, such as rRNAs, tRNAs, snoRNAs and snRNA. The remaining reads were used for further analyses.

### Identification of known and novel porcine miRNAs

**Fig 3:**
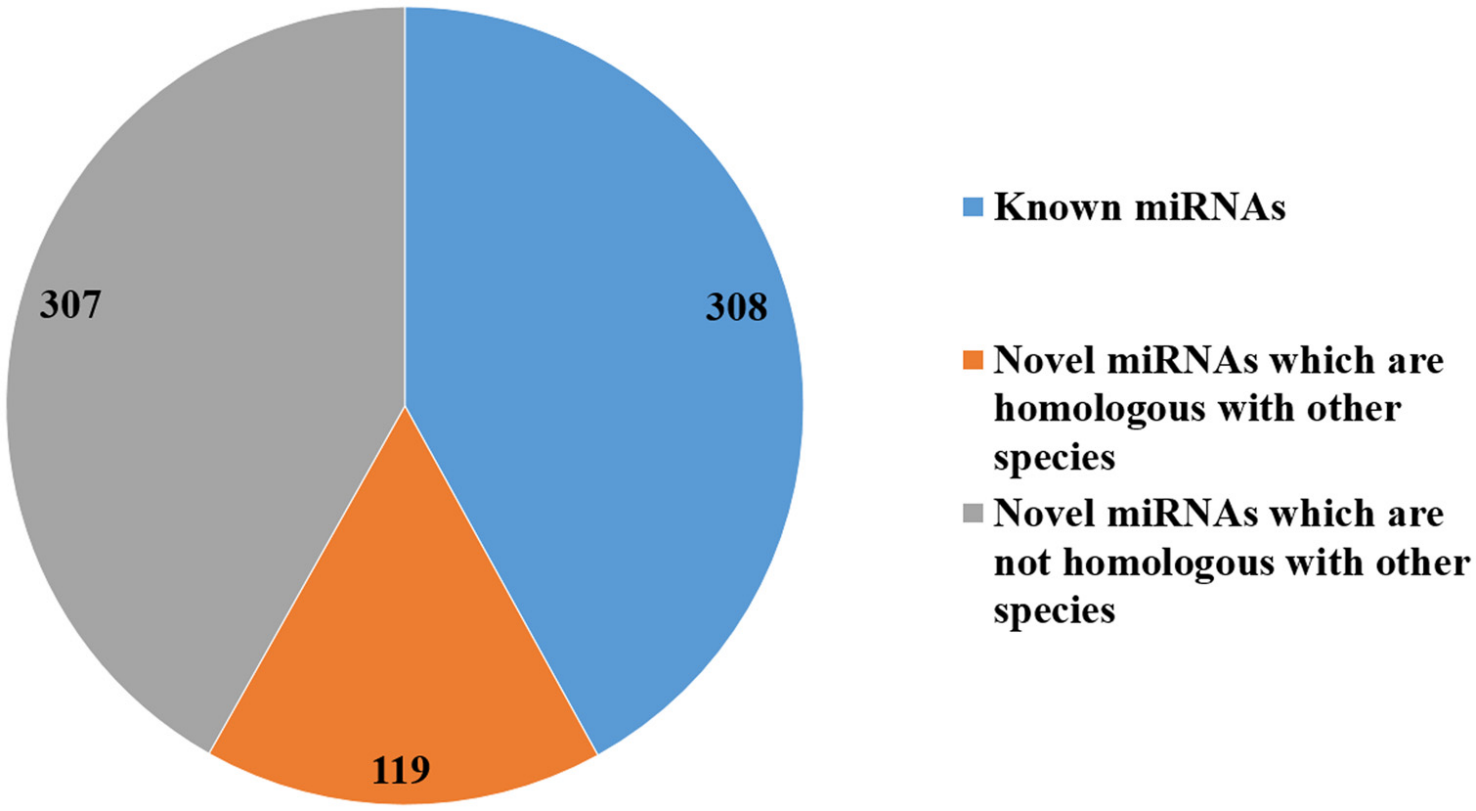
Number of known miRNAs, homologous novel miRNAs, and nonhomologous miRNAs.

A total of 411 mature porcine miRNAs are annotated in the miRBase database [23]. The homology search detected 308 known miRNAs (S1 Table) and 426 novel miRNAs (named ssc-miR-new-N, N = 1–426; S2 Table) in the ten sRNA libraries. Among the 426 novel miRNAs, 119 were homologous with miRNAs from other species, and no homologs were found for the remaining 307 (Fig 3). The known and novel miRNAs together comprised about 93.7 M reads, corresponding to 44% of the total reads in the ten sRNA libraries (Fig 1).

### Characterization of miRNAomes in porcine skeletal muscle at different postnatal stages

**Fig 4:**
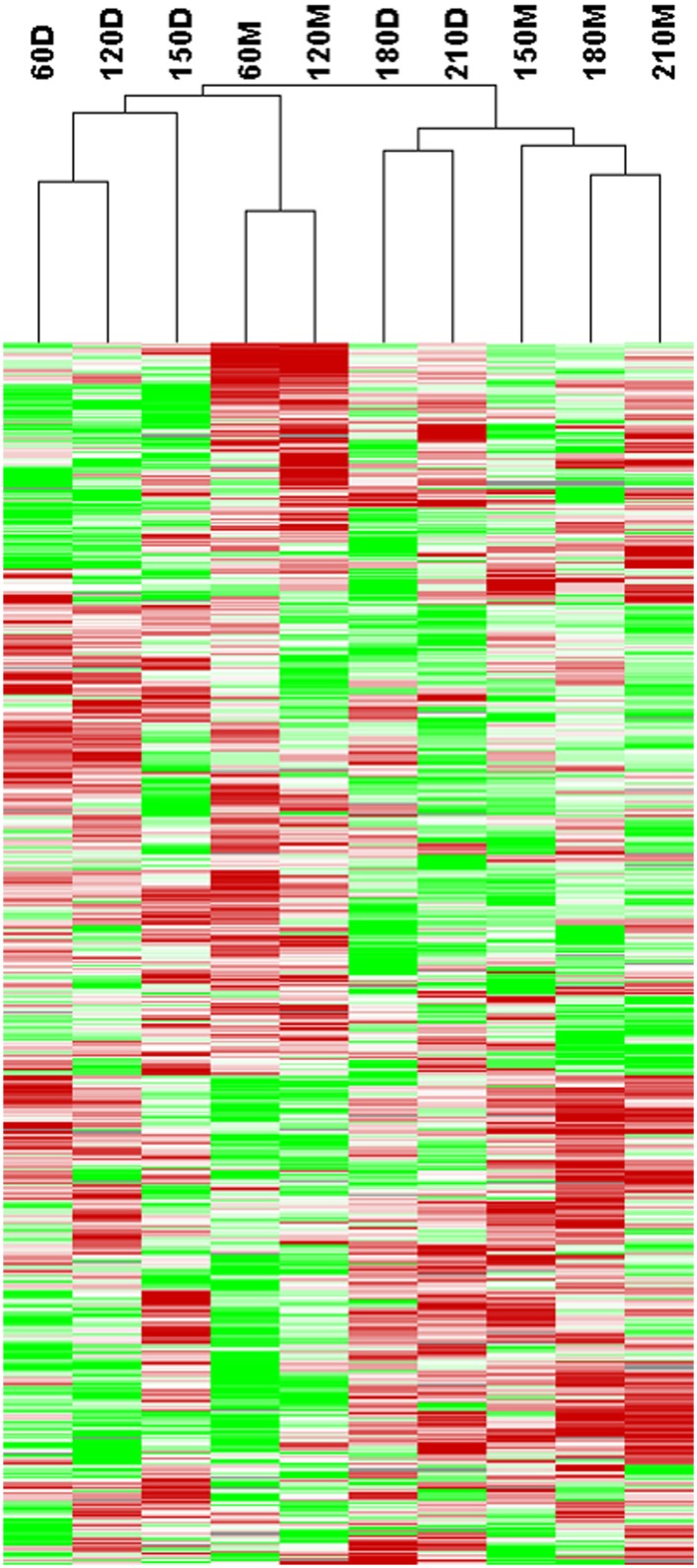
Cluster analysis of miRNA libraries from skeletal muscle of Large White and Min pigs. The skeletal muscle was collected at different developmental stages. LW, Large White pig; M, Min pig.

Systematic cluster analysis was performed to compare the relationships among the miRNAs in the ten skeletal muscle libraries. We found that the miRNAs in the ten libraries clustered first by the development stage and then by the breed (Fig 4). For example, miRNAs expressed in the *longissimus dorsi* muscles at the early developmental stages, such as 60 and 120 dpn, were grouped more closely. These results showed that the expressions of miRNAs have greater temporal differences than that between two breeds.

Almost all known myomiRs were identified in the analyzed muscle samples. The final miRNA expression values were calculated as TP5M (transcripts per 5 million). The most abundant miRNA was ssc-miR-206, which was present by more than 4,600,000 TP5M in ten libraries. Ssc-miR-1 and ssc-miR-133 were also highly expressed and ranked the 4^th^ and 28^th^ respectively. Two miRNAs, ssc-miR-26a [29, 30] and ssc-miR-378 [31], that ranked 2^nd^ and 3^rd^, respectively, have been reported to be involved in the proliferation and differentiation processes of skeletal muscle. Most novel miRNAs had an expression value below 100 TP5M, which were less abundant than the known miRNAs. However, several novel miRNAs had a higher expression level. For example, ssc-miR-new-371 and ssc-miR-new-108 were ranked 21^st^ and 30^th^. Ssc-miR-new-371 and ssc-miR-new-108 were homologous to hsa-miR-381-3p and hsa-miR-128-3p, both of which have been shown to be related to skeletal muscle development [32, 33]. However, some of the novel miRNAs with high abundance (e.g., ssc-miR-new-37 and ssc-miR-new-56) did not find homologs from other species, and their functions in skeletal muscle needed to be further clarified.

### Differentially expressed miRNAs between LW and M pig

**Fig 5:**
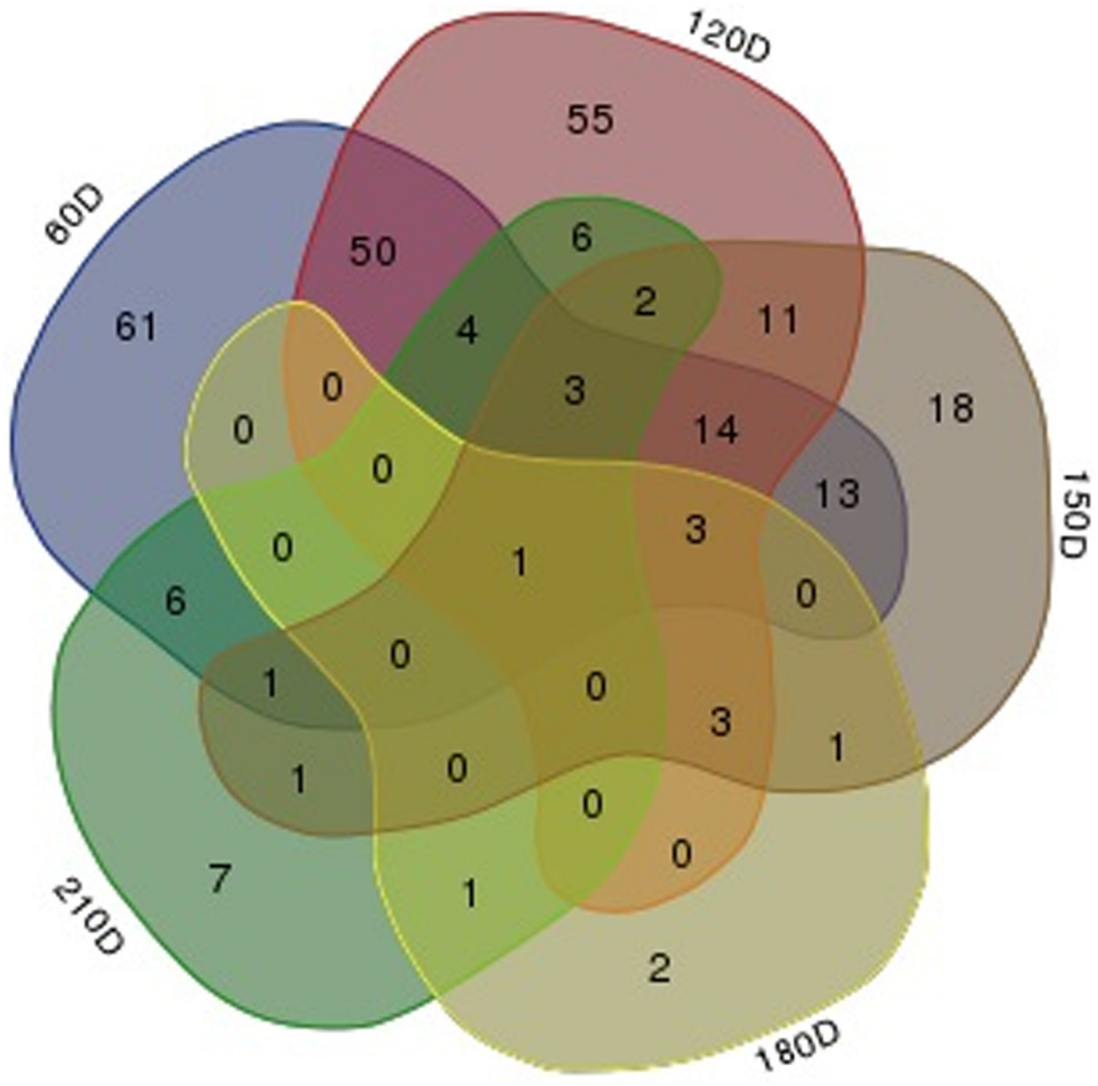
Pairwise comparisons of differentially expressed (DE) miRNAs in skeletal muscle at different developmental stages. The comparisons were made for Large White and Min pigs. The numbers marked in the overlapping areas indicate the common DE miRNAs.

**Fig 6:**
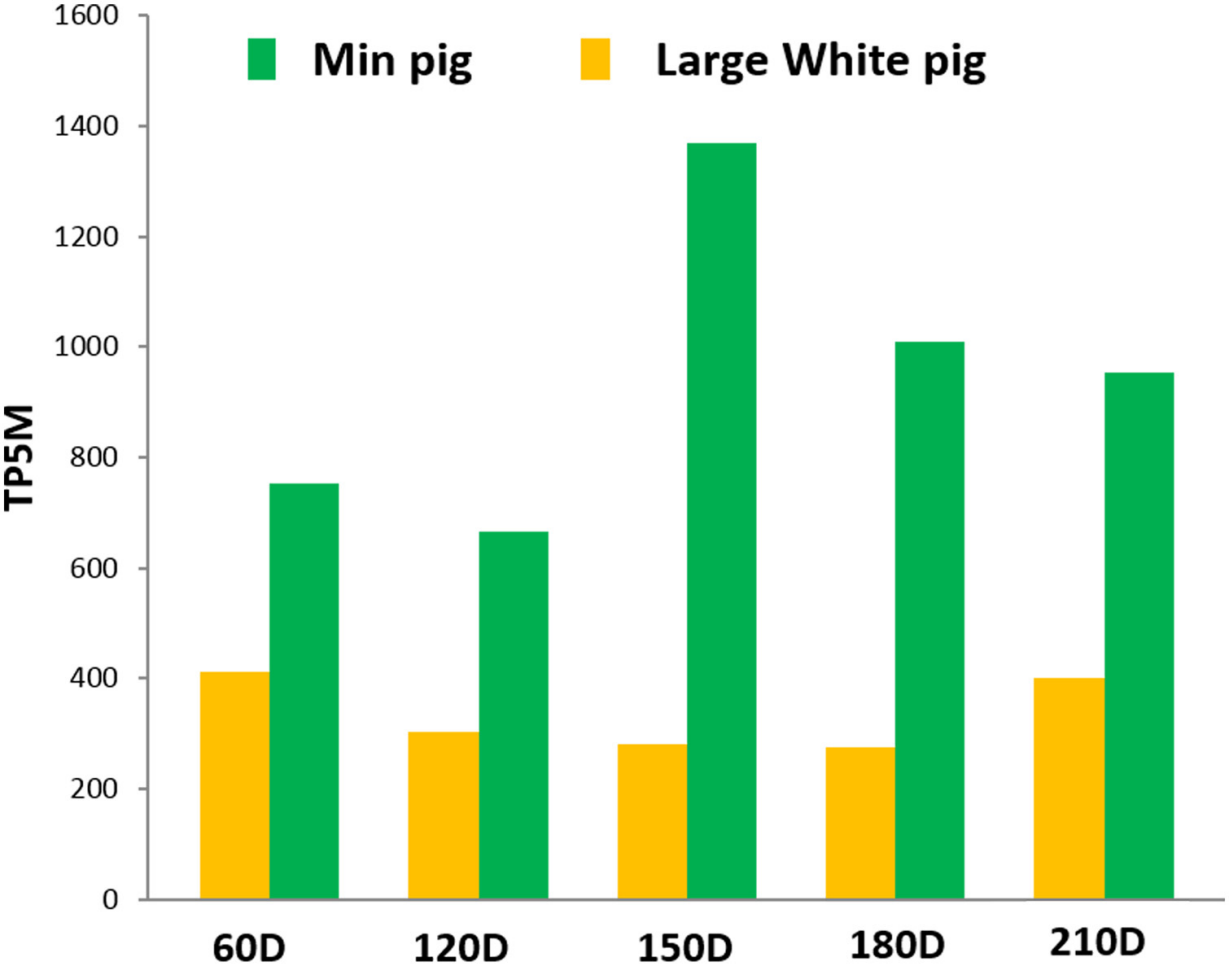
Expression levels of miR-204 at five postnatal development stages between Large White and Min pigs.

To identify miRNAs related to differences of postnatal muscle hypertrophy between LW and M pigs, we performed pairwise comparisons of 734 miRNAs (308 known and 426 novel) between two breeds at each developmental stage. We detected 154, 148, 70, 11, and 31 miRNAs that were differentially expressed (*P*-value <0.01) at 60, 120, 150, 180, and 210 dpn, respectively, between two breeds (Fig 5). The numbers of DE miRNAs at 60 and 120 dpn were larger than that at the later development stages. Overall, the expression of 263 miRNAs was significantly altered between two breeds at one or more developmental stages (S3 Table). Notably, ssc-miR-204, which was detected in all ten libraries, was significantly more highly expressed (*P*-value <0.01) in M compared with LW at all five developmental stages (S4 Table and Fig 6).

### MicroRNA targets prediction, GO and KEGG pathway analyses

To determine the biological function of ssc-miR-204, we obtained 554 predicted target mRNAs using TargetScan [26]. GO analysis identified 45 target genes that were enriched in cell proliferation, including Janus kinase 2 (*JAK2*) [34], SRY (sex determining region Y)-box 4 (*SOX*4) [35], insulin-like growth factor binding protein 5 (*IGFBP5*) [36], cyclin D2 (*Ccnd2*) [37], and sirtuin 1 (*SIRT1*) [38], all of which have been reported previously as the targets of miR-204 and involved in the regulation of cell proliferation. Another enriched GO category was cellular biosynthetic process, including disheveled segment polarity protein 3 (*DVL3*), which was shown previously to be modulated by miR-204 to promote the adipogenic differentiation [39] (S5 Table, S1 Fig). The KEGG pathway analysis identified 81 pathways that were enriched for the target genes. The most overrepresented targets belonged to the mitogen-activated protein kinase 1(MAPK), Wnt, Janus kinase-signal transducer and activator of transcription (Jak-STAT), and transforming growth factor-beta (TGF-beta) signaling pathways (S6 Table, S2 Fig).

### Differentially expressed miRNAs among different developmental stages

DE miRNAs between two adjacent developmental stages were detected by comparing the expression of 734 identified miRNAs in two breeds. We identified 71, 96, 69, and 30 DE miRNAs (*P*-value <0.01) in the 60/120, 120/150, 150/180, and 180/210 dpn comparisons, respectively, in LW pig (Table 1 and S7 Table), among which four miRNAs (ssc-miR-127, ssc-miR-331-3p, ssc-miR-new-304, and ssc-miR-345-3p) were common (S3 Fig and S7 Table). In addition, we identified 199, 127, 140, and 37 DE miRNAs in the 60/120, 120/150, 150/180, and 180/210 dpn comparisons, respectively, in M pig, among which nine miRNAs (ssc-miR-23a, ssc-miR-new-276, ssc-miR-142-3p, ssc-miR-142-5p, ssc-miR-499-5p, ssc-miR-15a, ssc-miR-new-386, ssc-miR-new-421, and ssc-miR-144) were common (S4 Fig and S7 Table).

**Table 1:** Differentially expressed miRNAs between every two adjacent developmental stages in Large White and Min pigs.

Pairwise comparisons^a^	Total DE^b^ miRNAs	Up-regulated miRNAs	Down-regulated miRNAs
LW 60/120	71	41	30
LW 120/150	96	47	49
LW 150/180	69	24	45
LW 180/210	30	17	13
M 60/120	199	98	101
M 120/150	127	69	58
M 150/180	140	57	83
M 180/210	37	20	17

^a^LW, Large White pig; M, Min pig.

^b^DE, differentially expressed (*P*-value <0.01).

### MicroRNA targets prediction, GO and KEGG pathway analyses

To determine the biological functions of DE miRNAs (*P*-value <0.01) during skeletal muscle postnatal development in LW and M pigs, the target mRNAs of the four (LW) and nine (M) miRNAs that were differently expressed between every adjacent developmental stages, were predicted by TargetScan [26] and miRDB [27]. A total of 696 (LW) and 5329 (M) predicted targets were obtained. In both breeds, the main GO categories assigned to the target genes were related to transcription, metabolic and biosynthetic processes (S5 Table). The KEGG pathway analysis showed that the targets of four DE miRNAs in LW were involved in the MAPK and Wnt signaling pathways (S6 Table), while the targets of nine DE miRNAs in M were involved in the MAPK and Wnt signaling pathways, as well as the TGF- beta signaling pathway.

## Discussion

The molecular mechanism regulated by miRNAs that underlies the regulation of skeletal muscle postnatal hypertrophy is complex and remains unclear. In this study, we analyzed the miRNAomes of *longissimus dorsi* muscle collected at five postnatal stages (60,120,150,180,210 dpn) in LW and M by Illumina sequencing.

Highly abundant miRNAs may play important roles in the regulation of skeletal muscle development. Hou et al. [40] showed that ssc-miR-206, ssc-miR-378, and ssc-miR-1 were expressed at extremely high levels in the *longissimus dorsi* muscles of Tong Cheng pigs. In the present study, we found that ssc-miR-206, ssc-miR-378, and ssc-miR-1 were ranked 1^st^, 3^rd^, and 4^th^ in abundance among the ten libraries, which is consistent with the previous study. However, some of the results from two studies were different. For example, we found that the expressions of ssc-miR-26a and ssc-miR-125b were much higher than their expressions reported in the previous study [40]. These differences in abundance levels may be due in part to the different developmental stages used in two studies. Hou et al. [40] identified miRNAs from a series of developmental stages, including post-gestation period and postpartum period, while our study was focused only on postnatal stage. Thus, ssc-miR-26a and ssc-miR-125b may play vital roles in the regulation of skeletal muscle postnatal hypertrophy.

The pairwise comparisons at each developmental stage in two breeds, showed that the number of DE miRNAs was largest at 60 dpn. Some reports have suggested that there may be a transition of slow-oxidative to fast-glycolytic fiber types from birth until 60 dpn [41], and the cross-section area of fast-glycolytic fiber was larger than that of slow-oxidative fiber [42]. Thus, we speculate that the DE miRNAs at 60 dpn may play a role in the fiber type transition to improve hypertrophy. In addition, Lee [43] reported that the major increase in intramuscular fat began after 16 weeks of age in pig. Our results showed that at 120 dpn the difference in abundance of DE miRNAs was larger than that at later development stages, suggesting that this time point may be important for intramuscular fat accumulation controlled by the DE miRNAs.

Our results also showed that the differences in the miRNAomes were smaller at 180 and 210 dpn between LW and M, with only 11 and 31 DE genes, while at 60, 120 and 150 dpn there were 154, 148, and 70 DE miRNAs, respectively. Further, the identification of DE miRNAs between two adjacent developmental stages in two breeds also showed that there were fewer DE miRNAs between 180 and 210 dpn than that between earlier stages (from 60 to 180 dpn). These results are consistent with previous studies, which found that muscle fiber continued to mature until 180 dpn [2, 18, 44, 45].

We hypothesized that the DE miRNAs between LW and M pigs are likely to be involved in the regulation of skeletal muscle hypertrophy. In this study, a total of 263 DE miRNAs between two breeds were identified at one or more developmental stages, among them ssc-miR-204 was common. MiR-204 had been shown to inhibit cell proliferation in various types of cell. For example, overexpression of hsa-miR-204 inhibited the proliferation and promoted the apoptosis in breast cancer cells by targeting *JAK2* [34]. In renal cell carcinoma, hsa-miR-204 directly targeted *SOX4* to inhibit cell proliferation, migration, and invasion [35]. Moreover, hsa-miRNA-204 inhibition was reported to promote human cardiomyocyte progenitor cells (hCMPC) proliferation, and hsa-miRNA-204 was required for hCMPC differentiation by its target, the activating transcription factor *ATF-2* [46]. Our results showed that ssc-miRNA-204 was significantly more highly expressed (*P*-value <0.01) in M compared with LW pigs at five postnatal developmental stages. Thus, we predicted that ssc-miR-204 inhibited skeletal muscle postnatal hypertrophy in M pig by controlling myoblast proliferation. The targets prediction and GO analysis identified 45 target genes related to cell proliferation including the genes mentioned above. However, the target genes of ssc-miR-204 and its role of regulation during the skeletal muscle postnatal development process need further studies.

MiR-204 may be also involved in intramuscular fat deposition. Hsa-miR-204-5p was predicted to play a role in insulin sensitivity and high-density lipoprotein (HDL) cholesterol metabolism by targeting the *ACACB* transcript in human adipose tissue [47]. In mice that were fed a high-fat diet, mmu-miR-204-5p was shown to be down-regulated in the adipose tissue [48]. In addition, the expression of ssc-miR-204 was reported to be higher in Chinese Meishan pigs than that in LW pigs [49]. In our study, an enriched GO category of the ssc-miR-204 target genes was cellular biosynthetic process, including disheveled segment polarity protein 3 (DVL3), which was previously reported to be modulated by hsa-miR-204 to promote adipogenic differentiation [39] (S5 Table, S1 Fig).

The KEGG pathway analysis identified 81 pathways that were enriched for the targets of miR-204. The most overrepresented targets belonged to the MAPK, Wnt, Jak-STAT, and TGF-beta signaling pathways (S6 Table, S2 Fig). The MAPK signaling pathway is closely related to the regulation of myogenesis [50] and inhibition of lipogenesis [51]. The Wnt signaling pathway regulates the homeostasis of adult muscle [52] and is known to be involved in adipogenesis metabolism. The Wnt/beta-catenin signaling pathway was reported to inhibit porcine adipogenic differentiation potential [53]. The Jak-STAT pathway was also found to play an important role in the regulatory mechanism of myogenic differentiation [54] and adipocyte differentiation [55] in human. Here, we focused on the TGF-beta signaling pathway because many studies have reported that the TGF-beta family played important roles in skeletal muscle development [56, 57]. Myostatin is a member of the TGF-beta superfamily and acts as a negative regulator of postnatal muscle growth [58]. In future studies, pathway analysis may add further insights into the vital roles played by miR-204 during skeletal muscle postnatal development.

Furthermore, DE miRNAs between two adjacent developmental stages in two breeds may be closely related to skeletal muscle hypertrophy. In M pig, ssc-miR-499 was differentially expressed in four comparison groups. In a previous study, hsa-miR-499 was associated with Duchenne muscular dystrophy and could serve as a promising biomarker for its diagnosis and disease progression [59]. Both ssc-miRNA-142-3p and ssc-miRNA-142-5p were also differentially expressed in several comparison groups in M pig, and down-regulation of these two miRNAs was found to be critical in cardiac hypertrophy [60]. In addition, mmu-miR-142-3p and mmu-miR-142-5p were shown to be up-regulated in adipose tissue during the development of obesity in mice [48]. Ssc-miR-331 was identified in the comparison groups in LW pig, and has been reported to be up-regulated during myogenic differentiation in human [61]. However, the exact roles of these miRNAs in skeletal muscle hypertrophy and intramuscular fat deposition are still unclear, and require further study.

## Conclusion

This study was carried out to identify DE miRNAs in the miRNAomes of *longissimus dorsi* muscle collected at five postnatal stages between Large White and Min pigs. Comparative analysis between two breeds suggested that 60 and 120 days after birth were important stages for skeletal muscle hypertrophy and intramuscular fat accumulation. In addition, we identified several potentially important miRNAs that may play regulatory roles in skeletal muscle postnatal hypertrophy and adipogenesis metabolism, especially ssc-miR-204. Together, the results of this research will contribute to understanding the mechanism underlying skeletal muscle hypertrophy modulated by miRNAs, which could provide valuable information for pork quality improvement and human myopathy.

## Supporting Information

S1 Fig GO analysis for the targets of miR-204. Top 20 categories were given on Y-axis and gene counts were given on X-axis.
(JPG)

S2 Fig Pathway analysis for the targets of miR-204. The pathways were given on Y-axis and gene counts were given on X-axis.
(JPG)

S3 Fig Comparison of differentially expressed (DE) miRNAs at five postnatal development stages in Large White pig. The number marked in the overlapping areas shows the common DE miRNAs.
(JPG)

S4 Fig Comparison of differentially expressed (DE) miRNAs at five postnatal development stages in Min pig. The number marked in the overlapping areas shows the common DE miRNAs.
(JPG)

S1 Table Expression profiles of known miRNAs during swine skeletal muscle postnatal development (TP5M). LW, Large White pig; M, Min pig.
(XLS)

S2 Table Information and expression profiles of novel miRNAs in skeletal muscle of Large White pig and Min pig during postnatal development (TP5M). (XLSX)

S3 Table Differential expressed miRNAs in skeletal muscle between LW pig and M pig at each developmental stage. (XLSX)

S4 Table Differential expressed miRNAs in every intersection of five developmental stages between Large White and Min pig. (XLSX)

S5 Table GO categories of target genes of miR-204, 4 differently expressed miRNAs at all postnatal development stages in Large White pig and 9 differently expressed miRNAs at all postnatal development stages in Min pig. (XLS)

S6 Table KEGG pathway analysis for the targets of miR-204, 4 differently expressed miRNAs at all postnatal development stages in Large White pig and 9 differently expressed miRNAs at all postnatal development stages in Min pig. (XLS)

S7 Table Differentially expressed miRNAs between every two adjacent development stages in Large White pig and Min pig. (XLS)
