# Supplementary figures and images for: Comparative Analyses between Skeletal Muscle miRNAomes from Large White and Min Pigs Revealed MicroRNAs Associated with Postnatal Muscle Hypertrophy
Source: PLoS One. 2016 Jun 2;11(6):e0156780. doi: 10.1371/journal.pone.0156780 (PMC4890935; doi:10.1371/journal.pone.0156780)

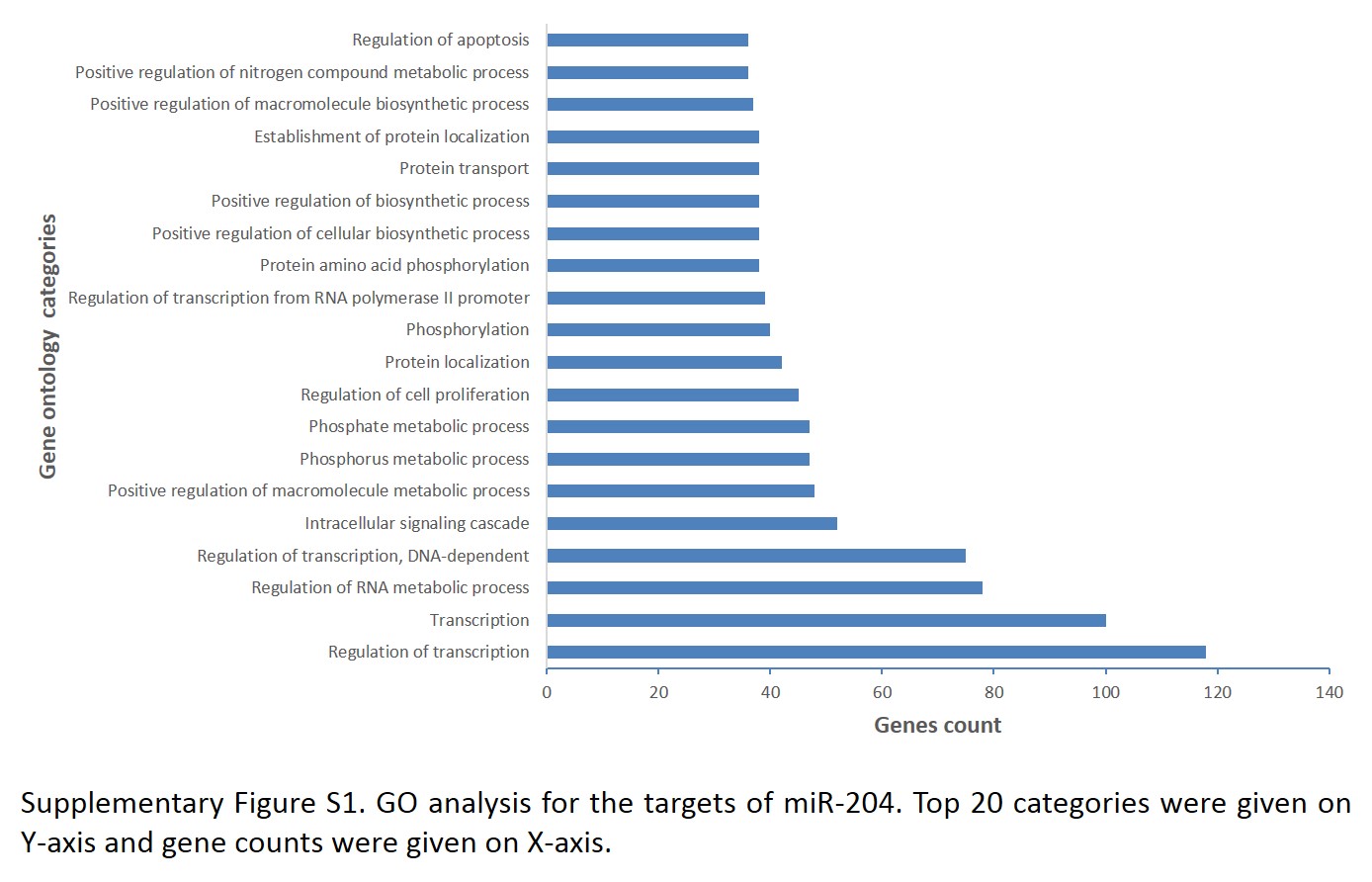

Supplement: S1 Fig — Top 20 categories were given on Y-axis and gene counts were given on X-axis. (JPG) [file pone.0156780.s001.jpg]

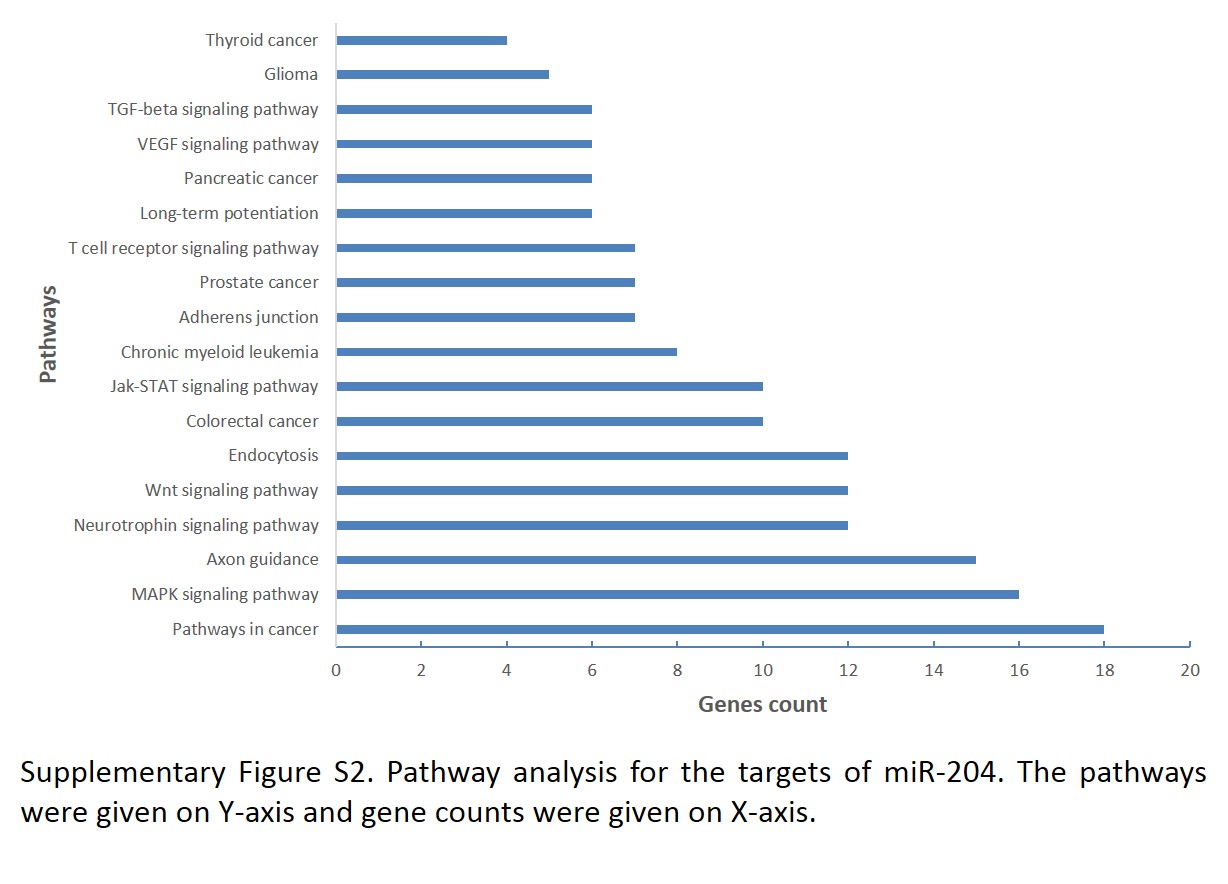

Supplement: S2 Fig — The pathways were given on Y-axis and gene counts were given on X-axis. (JPG) [file pone.0156780.s002.jpg]

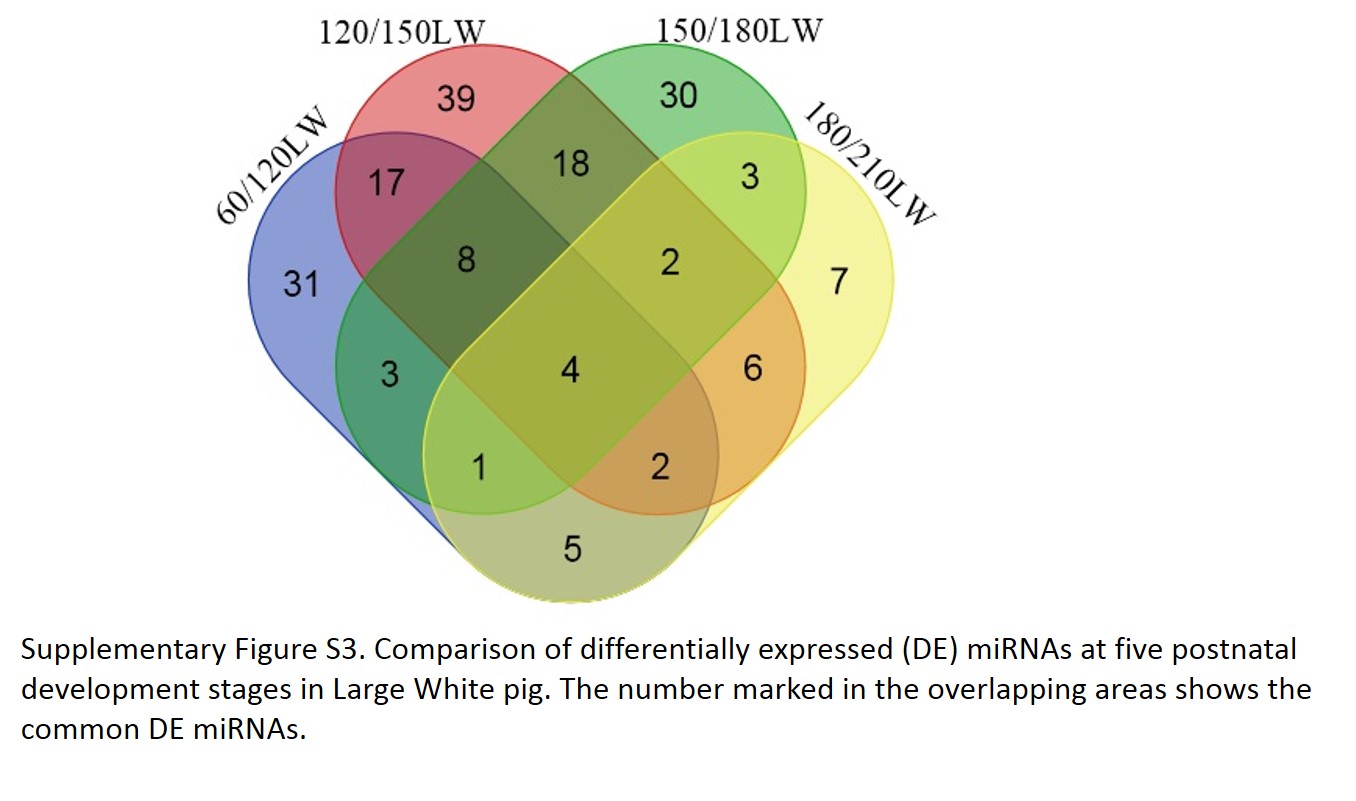

Supplement: S3 Fig — The number marked in the overlapping areas shows the common DE miRNAs. (JPG) [file pone.0156780.s003.jpg]

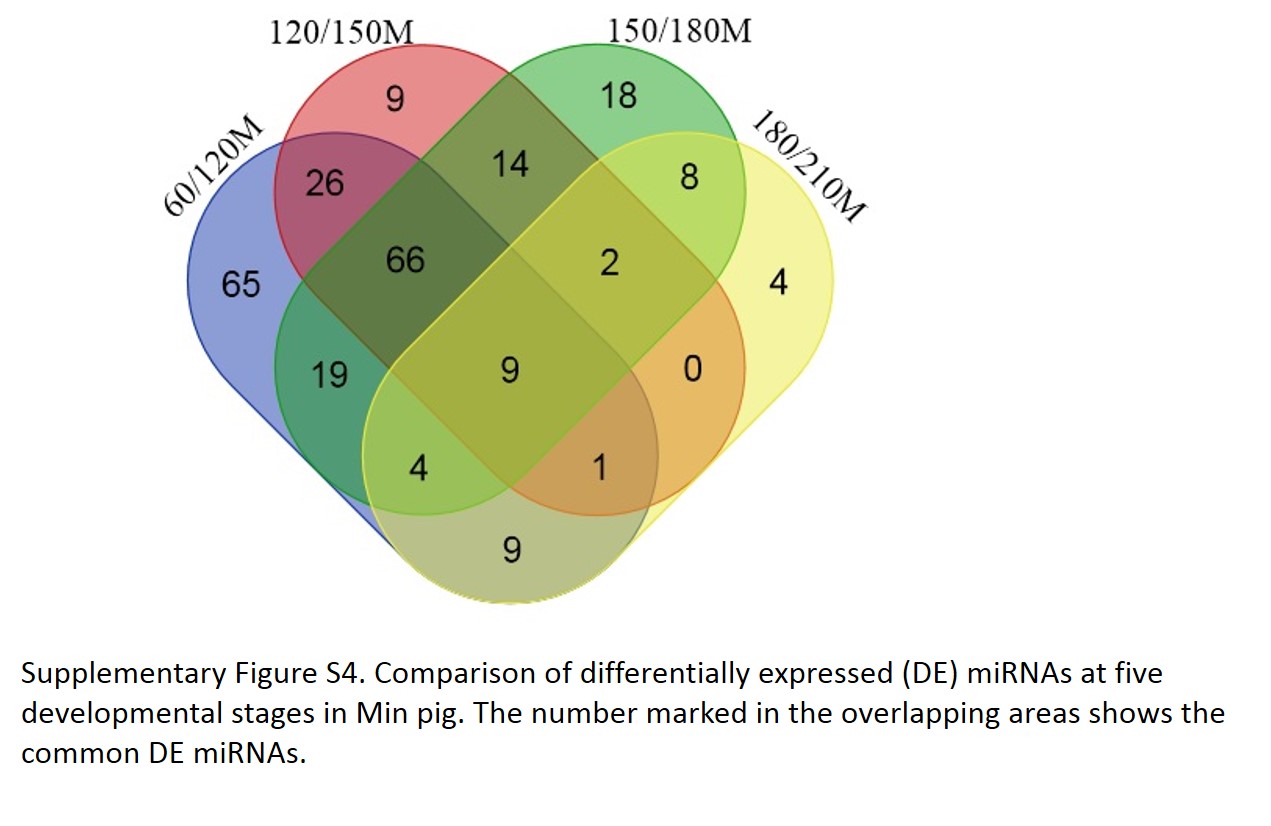

Supplement: S4 Fig — The number marked in the overlapping areas shows the common DE miRNAs. (JPG) [file pone.0156780.s004.jpg]
